# “Happiness tree”: a thematic art-based group counseling method for enhancing positive psychological traits in Chinese college students

**DOI:** 10.3389/fpsyg.2025.1651008

**Published:** 2025-08-20

**Authors:** Fangzhou Xie, Mengyao Tang, Shanshan Wu, Yong Wang

**Affiliations:** ^1^Student Mental Health Education Center, Hefei Preschool Education College, Hefei, China; ^2^Department of Student Affairs, Anhui Institute of International Business, Hefei, China; ^3^Department of Student Affairs, Hefei Economic and Trade Tourism School, Hefei, China

**Keywords:** art therapy, group counseling, college students, positive psychological traits, subjective well-being, self-efficacy

## Abstract

**Introduction:**

This study was a pilot study aimed to evaluate the effectiveness of a new counseling method “Happiness Tree” thematic art-based group counseling.

**Methods:**

We conducted an intervention study in which 36 psychologically healthy college students were instructed to create symbolic tree drawings and engage in reflective writing, helping them identify personal strengths and emotional resources. Students in the experimental group received six online group counseling sessions, while students in the control group received no intervention. Subjective well-being (SWB) and general self-efficacy (GSE) were measured before and after the intervention.

**Results:**

The results showed that participants in the experimental group experienced preliminary improvements in both SWB and GSE compared to the control group, suggesting potential benefits of the intervention.

**Discussion:**

These findings suggest that the “Happiness Tree” thematic art-based group counseling shows promise for cultivating positive psychological traits in young adults. Our research provides a useful tool for promoting mental health in educational settings.

## 1 Introduction

The mental health of college students has emerged as a pressing societal concern, with mounting pressures from academic demands, career uncertainty, familial expectations lead to psychological strain (Ning et al., 2024). According to the 2023 National Report on Mental Health Development of Chinese Citizens, 21.48% of college student were at risk of depression and 45.28% at risk of anxiety—both significantly higher than the rates observed in the general population. Therefore, improving mental health and cultivating positive psychological traits among college students have become pressing objectives for higher education institutions.

Positive psychology focuses on positive experiences, personal strengths, and the development of human potential and virtues. Its central goal is to promote happiness and personal growth (Seligman and Csikszentmihalyi, 2000). Positive psychology advocates that psychology should not only focus on alleviating suffering but also emphasize the study and cultivation of positive qualities and states. Early research focused on happiness and positive emotions, designing various interventions to enhance happiness and reduce depression (Seligman, 2004). In recent research, the focus of positive psychology gradually expanded from an individual perspective to include group well-being and cross-cultural adaptability. Scholars began to explore how happiness is embedded within social, cultural, and ecological systems, providing more comprehensive theoretical and practical guidance for mental health interventions (Lomas et al., 2021).

## 2 Literature review

### 2.1 Overview of positive psychological traits

Positive psychology emphasizes positive traits, and happiness can come from fulfilling experiences and self-transcendence through the practice of virtues (Donaldson et al., 2014). Positive psychological traits are stable characteristics shaped by the interaction of genetic predispositions and social experiences. They include positive personality traits, adaptive self-regulation, and positive emotional experiences like hope, optimism, and gratitude. These traits are essential for happiness and serve as key resources for enhancing self-efficacy, psychological resilience, and emotional regulation (Parkinson et al., 2023; Seligman, 2011). Seligman and Csikszentmihalyi (2000) also introduced the concept of “positive personal traits,” which includes characteristics such as love and vocation, courage, and interpersonal skills, highlighting their importance for enhancing individual happiness and mental health. Seligman (2004) viewed positive psychological traits as “virtues and strengths,” and suggested that these traits may improve psychological resilience and overall well-being. Fredrickson (2001) argued that positive psychological traits are crucial for accumulating psychological resources that enhance mental health and social adaptability. Recent empirical research supports these views, showing that traits such as gratitude, bravery, perseverance, and self-regulation are significant predictors of psychological health and well-being (Kaynak, 2025; Malik et al., 2025; Sheikh and Siddiqui, 2023). Building on these points, Peterson (2004) classified positive psychological traits into six core virtues including wisdom and knowledge, courage, love and humanity, justice, temperance, spirituality and transcendence, along with 24 character strengths.

#### 2.1.1 Subjective well-being and self-efficacy

Both subjective well-being and self-efficacy are often regarded as important indicators of positive psychological traits.

Subjective well-being (SWB) refers to an individual’s overall evaluation of life quality based on personal standards and their subjective appraisal of life circumstances. It comprises both cognitive and emotional components: cognitive well-being is reflected in life satisfaction, while emotional well-being involves the experience of both positive and negative emotions (Busseri, 2024; Diener, 2000). Research has shown that subjective well-being is closely related to positive psychological traits (Winzer et al., 2021; Xu et al., 2023). Positive traits such as hope, vitality, gratitude, curiosity, and love are strongly associated with higher levels of subjective well-being (Carr et al., 2020; Casali and Feraco, 2024; Hausler et al., 2017; Seligman, 2011). This suggests that positive psychological traits are not only central to theoretical inquiry but also serve as practical tools for promoting well-being.

Self-efficacy, first proposed by (Bandura, 1977), refers to an individual’s belief in their ability to accomplish tasks or goals. People with high self-efficacy tend to believe in their strengths and limitations. They are more likely to face challenges and put in effort to overcome obstacles. This mindset fosters positive emotional experiences, helping individuals manage stress and adversity (Caprara et al., 2006; Mu et al., 2024). Research shows that self-efficacy is crucial for emotional regulation and psychological resilience (Aizava et al., 2024; Călinici et al., 2020; Martinez-Calderon et al., 2020). Furthermore, enhancing self-efficacy helps individuals maintain resilience under stress, which ultimately supports overall mental health (Gilar-Corbi et al., 2024; Muenchhausen et al., 2021; Wang et al., 2023). Recent empirical research has found that self-efficacy is positively associated with multiple positive psychological traits in college students, such as hope and resilience (Guo et al., 2023). In short, self-efficacy not only lead to better performance but also promotes positive psychological traits.

#### 2.1.2 Strategies for enhancing positive psychological traits

Positive psychological traits can be cultivated through interventions and educational programs. Extensive research has explored strategies for enhancing these traits.

Positive Psychology Interventions (PPIs) are key approaches for enhancing positive psychological traits. These interventions have consistently been shown to improve subjective well-being, mental health, and social adaptability. Intervention studies have proved effectiveness of interventions such as “Three Good Things” and “Using Personal Strengths in a New Way.” These interventions have been shown to significantly enhance well-being and reduce depressive symptoms (Mongrain and Anselmo-Matthews, 2012; Seligman et al., 2005). DeBiase et al. (2021) found that PPIs significantly increased adolescents’ life satisfaction and classroom behavior. Studies have demonstrated that online PPIs have the potential to improve well-being and lessen depressive symptoms across diverse populations (Auyeung and Mo, 2018; Francis et al., 2021).

Another method for enhancing positive psychological traits is mindfulness training. According to Bolier et al. (2013), this method can improve emotional regulation, enhance subjective well-being, and reduce anxiety and depression. It has been widely applied to university students and adolescent populations. Research shows that cultivating traits such as optimism and self-efficacy helps college students better cope with life challenges, alleviate anxiety, and improve academic performance (Denovan and Macaskill, 2016). Mindfulness training helps students enhance self-awareness, improve psychological adaptability and social functioning, and strengthen their positive psychological traits. Positive psychology courses are also an effective approach for enhancing positive psychological traits. Studies have shown that these courses significantly improve life satisfaction, emotional stability, and subjective well-being among healthcare workers and students (Hobbs et al., 2022; Shaghaghi et al., 2019). Furthermore, online positive psychology courses have demonstrated comparable outcomes, significantly improving mental health and well-being (McDonald and Nanni, 2023).

Group counseling is a practical and engaging intervention. Studies on adolescent students have shown that positive psychology group counseling notably enhances self-esteem, self-efficacy, psychological resilience, and overall mental health, while reducing anxiety, depression, and interpersonal sensitivity (Kounenou et al., 2022; Shoshani and Steinmetz, 2014; Tan, 2023).

Among the various strategies for enhancing positive psychological traits, art therapy has gained increasing attention as an innovative intervention. Research indicates that art therapy provides emotional support through artistic expression, so it can improve mental health, enhance well-being, and facilitate emotional regulation and stress management (Samuel et al., 2021; Zhang et al., 2024).

### 2.2 Overview of art therapy

Art therapy is a therapeutic intervention that promotes mental health and emotional regulation through artistic creation. This therapy offers individuals a channel for emotional expression and self-exploration, helping them express their inner thoughts and emotions, resolve emotional conflicts, and restore psychological balance in a safe environment (Bozcuk et al., 2017). For instance, research has shown that this method can alleviate stress, reduce psychological distress, improve concentration, and enhance overall well-being (Stuckey and Nobel, 2010).

Art therapy originated in the early 20th century, when psychologists began exploring the integration of art into psychotherapy. Carl Jung established foundational practices by systematically employing artistic creation as an analytical tool. He believed that artistic creation could symbolize unconscious imagery and visualize dream content, thereby facilitating understanding of an individual’s inner world. Modern neuroscience research provides support for his view by demonstrating that artistic creation engages brain networks involved in imagination, memory, and emotion, such as the default mode network (DMN), which are associated with self-reflection and symbolic processing (Carroll, 2020; Prasad, 2022; Vaisvaser et al., 2024).

Over time, art therapy evolved into an independent discipline. Naumburg (1953) proposed that artistic creation serves as a form of symbolic communication, allowing direct expression of unconscious content. Engaging in spontaneous image creation enables individuals to experience free association, which facilitates emotional release and psychological healing. Kramer emphasized the therapeutic value of the art-making process itself, arguing that the process is more important than the final interpretation (Thompson, 2014). She introduced the concept of “art as therapy,” suggesting that through artistic creation, patients can transform unconscious conflicts into creative outcomes, thereby enhancing their mental health. In recent years, advances in neuroscience have highlighted the impact of art creation on brain activity, such as activation of the prefrontal cortex and reward systems, which enhance self-regulation and emotional control, thereby providing scientific support for art therapy (Bolwerk et al., 2014).

#### 2.2.1 Theoretical basis of art therapy: psychological and neuroscientific perspectives

Art therapy is theoretically rooted in psychoanalytic therapy. Projection and sublimation are defense mechanisms proposed by Sigmund Freud within the framework of psychoanalysis. Projection refers to the psychological process by which individuals attribute unacceptable conflicts, emotions, or desires to external objects or others. In art therapy, this allows subconscious material to be externalized. Through color, imagery, and spatial composition, individuals symbolically express inner conflicts, offering valuable insights for psychological analysis and therapeutic intervention.

Sublimation, on the other hand, involves transforming instinctual impulses (such as aggression or sexual drives) into socially acceptable and creative activities. As a constructive and adaptive defense mechanism, sublimation is regarded as a hallmark of psychological health and a defining feature of mature personality development. Artistic creation is widely regarded as a classic form of sublimation, enabling individuals to convert internal conflicts into meaningful and acceptable artistic expressions, thereby achieving emotional relief and psychological integration. In this process, the act of painting becomes central to the therapeutic experience.

While these concepts originate from classical psychodynamic theory, recent neuroscientific studies have begun to provide biological support for these mechanisms. For instance, Sambuco (2024) demonstrated that emotionally salient autobiographical recall activates the amygdala and ventromedial prefrontal cortex (vmPFC), supporting the transformation of raw affective signals into coherent symbolic representations. This process mirrors the symbolic transformation described in projection and sublimation, offering neural validation for their therapeutic relevance.

In a complementary line of research, early neuroscientific studies investigating the biological basis of art therapy drew upon the theory of cerebral lateralization, which posits functional specialization between the two hemispheres—with the right hemisphere predominantly involved in visuospatial perception, emotional processing, and creativity (Gazzaniga, 2000; Sperry, 1968). Art therapy has been shown to activate neural networks associated with emotional processing and creative expression, particularly within the limbic system and prefrontal cortex (Chatterjee, 2011). It engages the dynamic interaction of multiple brain systems, especially those associated with emotion regulation and cognitive control. Neuroimaging studies reveal that artistic creation enhances functional connectivity between the prefrontal cortex and subcortical limbic structures, thereby improving cross-regional communication (Deshmukh et al., 2018; Kaimal et al., 2016; King and Parada, 2021; Strang, 2024). This neural synergy facilitates the integration of emotional and cognitive processes, ultimately enhancing overall mental health.”

#### 2.2.2 Applications of art therapy

As a non-verbal psychological intervention, art therapy has proven effective across a wide range of populations, including children, adolescents, and individuals with psychological disorders, due to its unique ability to facilitate emotional expression and psychological regulation. For instance, in treating children and adolescents with post-traumatic stress disorder (PTSD), art therapy serves as an effective tool for expressing complex emotions, facilitating emotional release, and finding meaning (Pifalo, 2006). Ugurlu et al. (2016) confirmed that expressive therapies, like painting and music, significantly alleviate PTSD symptoms in children. A systematic review has also emphasized the benefits of art therapy in supporting children who have experienced trauma, particularly in improving mental health during psychological counseling (Braito et al., 2022). In addressing behavioral disorders, studies show that art therapy helps reduce behavioral and emotional dysregulation in children diagnosed with Oppositional Defiant Disorder (ODD), while promoting self-reflection (Khadar et al., 2013). Additionally, research involving children with learning disabilities suggests that art therapy enhances social adaptation and emotional self-regulation by encouraging emotional expression and cognitive exploration (Freilich and Shechtman, 2010). Among college students, art therapy is associated with a reduction in anxiety and depression, as well as improvements in self-awareness and overall well-being (Bazargan and Pakdaman, 2016; Mu et al., 2024; Sandmire et al., 2012).

Despite its well-documented benefits in emotional regulation and psychological intervention, art therapy has certain limitations regarding intervention specificity. Unstructured art-making often lacks clear goals when addressing complex psychological issues, which can lead to inconsistent therapeutic outcomes., Thus structured approaches have gained attention in recent years. Thematic art-making, which incorporates predefined themes, offers a framework for psychological intervention while preserving the flexibility of artistic expression. This method has proven particularly effective in promoting mental health among college students, fostering positive psychological traits like hope, self-confidence, and gratitude.

### 2.3 Overview of thematic art drawing

Thematic art drawing is a form of art therapy that uses specific themes to help individuals express and explore their inner world. It blends the flexibility of artistic expression with the focused nature of therapeutic intervention. This combination makes thematic art drawing a valuable extension of traditional art therapy within a more structured framework. The concept of thematic art drawing originates from classic projective drawing tests, such as the House-Tree-Person Test (HTP), Draw-a-Person Test (DAP), and Baum Test. These early tools were widely used in psychological assessments (Buck, 1948; Koch, 1952; Machover, 1949). Building upon this foundation, thematic art drawing has developed into a structured psychological treatment method. Its goal-directed design allows it to be an effective tool for psychological intervention and treatment.

In art therapy, thematic art drawing helps individuals address specific psychological challenges like trauma, anxiety, depression, and self-identity. Empirical research suggests that thematic art drawing enhances attentional focus, reduces internal psychological conflicts, and is more effective than free-form art in treating anxiety symptoms (Cross and Brown, 2019). Studies on chronic pain patients indicate that it helps reduce stress and negative emotions related to pain, strengthens psychological resilience, and promotes well-being (Hass-Cohen et al., 2021). Similarly, research on personality disorders shows that thematic art drawing improves self-awareness, facilitates emotional recognition, and adaptive emotional regulation strategies (Haeyen et al., 2018). For college students, projective drawing tests like the HTP are useful in identifying psychological distress and guiding targeted counseling interventions (Wang and Chen, 2018).

Thematic art drawing has exhibited significant potential in diverse populations and psychological contexts in recent years. While prior studies confirm that art-based interventions (e.g., painting) facilitate emotional regulation, coping strategies, and psychological well-being among adolescents (Bosgraaf et al., 2020; Stuckey and Nobel, 2010), empirical research on their efficacy in cultivating positive psychological traits in college students remains limited. To address this research gap, the present study introduces a structured thematic art-based intervention model known as the “Happiness Tree.”

### 2.4 The “happiness tree” model and research hypothesis

The “Happiness Tree” is a thematic art-based drawing intervention grounded in positive psychology and art therapy. It combines symbolic imagery, creative drawing, and reflective writing to help individuals identify and reinforce psychological resources related to happiness. With over a decade of empirical validation, the model uses the tree metaphor—comprising roots, trunk, and crown—to help participants explore their emotional strengths, social support, and future aspirations. Tree imagery was chosen for its simplicity and strong associations, making it more accessible than other forms of figure drawing (Gu et al., 2020). In psychological research, trees are commonly used as projective symbols that reflect emotional and cognitive states, social connection, and personal development. Different parts of the tree represent different emotional domains or life dimensions in the HTP test (Yu et al., 2016). Similarly, within symbolic drawings, trees often represent internal resilience, vitality, and potential. The tree metaphor encourages individuals to examine their personal histories, development, and aspirations in growth-oriented and self-actualization contexts.

The model consists of three core components:

(1) Roots: Representing intrinsic contributors to happiness, such as personality traits, hobbies, and physical health;
(2) Trunk: Denoting external support systems, including family, friends, and interpersonal relationships;
(3) Crown: Symbolizing past experiences of happiness and aspirations for the future.

The “Happiness Tree” aims to guide individuals in identifying and reinforcing their personal happiness resources. It fosters positive emotional experiences and helps strengthen positive psychological traits. According to the Broaden-and-Build Theory (Fredrickson, 2001), positive emotions expand cognitive and emotional capacities, which enables individuals to face challenges with more flexibility. The “Happiness Tree” operates as a mechanism that empowers individuals to externalize existing happiness resources through visual and written expression, thereby reinforcing positive emotions and broadening their cognitive perspectives.

Furthermore, artistic and written expression facilitate the concretization of both internal and external psychological resources, enhancing coherence between emotional expression and self-awareness. This process supports more effective emotional regulation, allowing individuals to recognize their emotional origins more clearly and cognitively reconstruct their happiness framework.

To assess and examine the feasibility and effectiveness of the “Happiness Tree” thematic art-based drawing in fostering positive psychological traits, this study is designed as a pilot exploratory investigation and utilizes subjective well-being and general self-efficacy as key indicators. Group-based interventions are employed to assess its impact on college students. Accordingly, this study hypothesizes that participation in the “Happiness Tree” thematic art-based group counseling program will significantly enhance positive psychological traits—specifically, subjective well-being and self-efficacy—among college students.

## 3 Materials and methods

### 3.1 Participants

**Figure 1:**
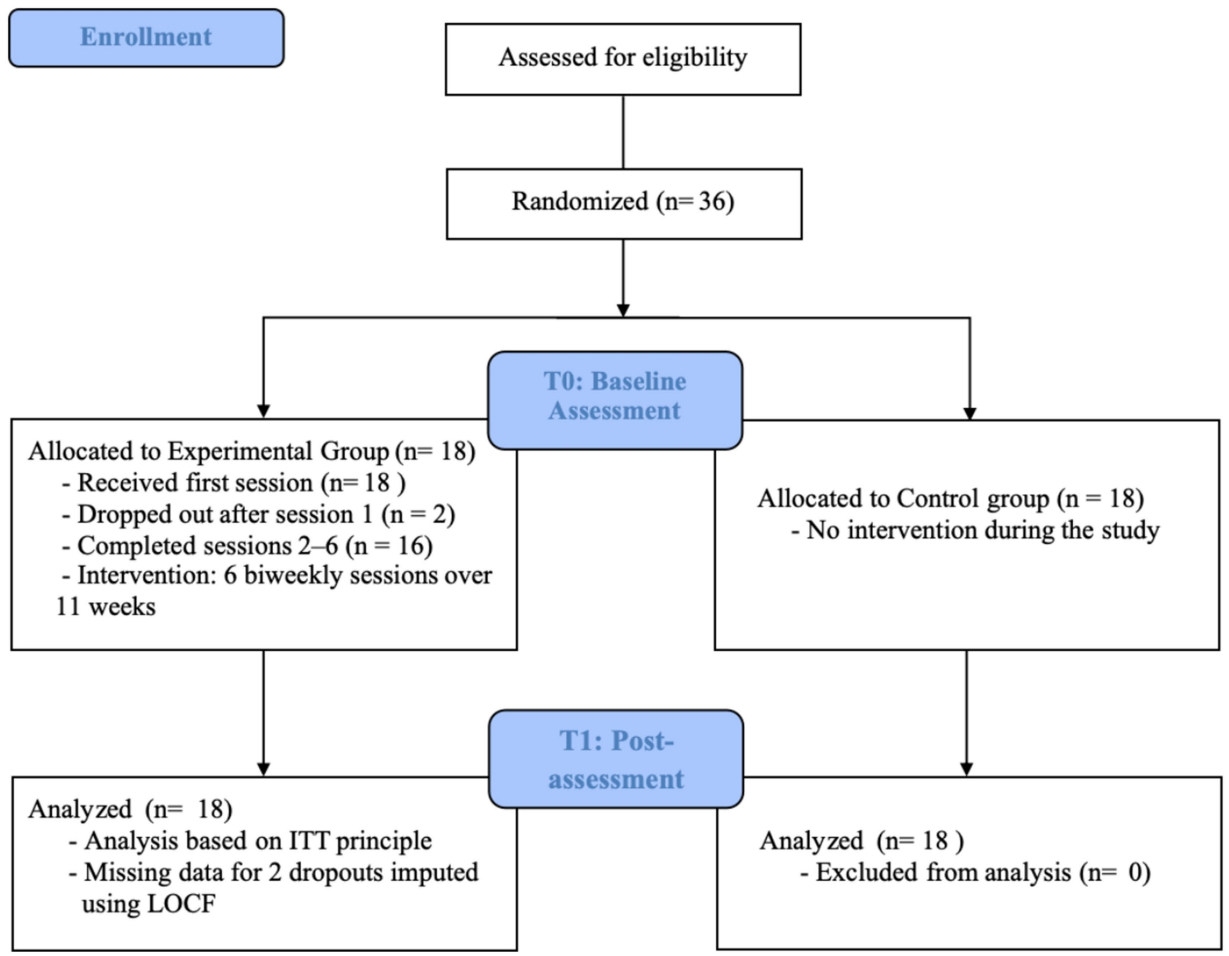
Flowchart of the study design.

A convenience sampling method was used to recruit participants from a public university in China and a total of 36 participants voluntarily participated in this study. Sample size was determined by practical considerations for this exploratory pilot study. No formal power calculation was conducted *a priori*. The results of the General Health Questionnaire (GHQ-20) (Murphy, 1973) indicated that all participants had good mental health. Participants were randomly assigned to groups using a computer-generated randomization sequence with allocation concealment. Allocation concealment was maintained until all baseline assessments were completed. Outcome data were collected by a trained student research assistant who was blinded to group assignment and not involved in the intervention process. Two participants dropped out due to inability to attend the full intervention. Ultimately, 16 participants (all female) were assigned to the experimental group, and 18 participants (2 male, 16 female) were assigned to the control group. All participants were first- or second-year students. The experimental group received six biweekly online group counseling sessions over 11 weeks, while the control group received no intervention during the study period. Two weeks after the experiment, the control group was offered a “Happiness Tree” group counseling experience. All participants gave written informed consent prior to participation. The study flow is illustrated in Figure 1.

### 3.2 Measurement instruments

The General Health Questionnaire (GHQ-20) was developed by Murphy (1973) and later revised into a 20-item Chinese version by Li and Kam (2002). The questionnaire includes three subscales: self-affirmation, depression, and anxiety. It uses a dichotomous (“Yes”/“No”) scoring system, where a “Yes” is scored 1 point and “No” is scored 0 points. Items 7 and 10 are reverse scored. In this study, the self-affirmation subscale scores were reverse-coded and included in the total score, where higher scores indicate poorer mental health. The internal consistency coefficient for this scale was 0.82.

The General Self-Efficacy Scale (GSES) was created by Schwarzer (1997) and translated into Chinese by Wang et al. (2001). The scale includes 10 items rated on a 4-point Likert scale, ranging from 1 (“Completely incorrect”) to 4 (“Completely correct”). Higher scores indicate higher self-efficacy. The internal consistency coefficient for this scale was 0.87.

Subjective well-being was assessed using the Satisfaction with Life Scale (SWLS) and the Positive and Negative Affect Schedule (PANAS). The SWLS, developed by Diener (2000), consisted of five items rated on a 7-point Likert scale, ranging from 1 (“Strongly Disagree”) to 7 (“Strongly Agree”). Higher scores reflect greater life satisfaction. The internal consistency coefficient for SWLS was 0.84.

The PANAS, developed by Watson et al. (1988) and revised by Qiu et al. (2008), includes 10 positive and 10 negative emotion words. Responses are rated on a 5-point scale from 1 (“None or very slight”) to 5 (“Very strong”). Higher scores indicate stronger positive or negative emotions. The internal consistency coefficient for PANAS was 0.83.

Following prior research (Liang et al., 2024), subjective well-being scores were calculated as the weighted average of life satisfaction scores, positive affect scores, and reverse-coded negative affect scores, with a weight ratio of 1.1:1:1.

### 3.3 Group counseling intervention

The experimental group received “Happiness Tree” thematic art-based group counseling, facilitated by two certified psychological counselors from the university. The program design and implementation were supervised by a psychology expert, who was consulted at each stage of the intervention to dynamically adjust counseling strategies and ensure the sessions’ effectiveness. The counseling program was theoretically grounded in positive psychology and designed based on the Balint group structure. It comprised six sessions, delivered in two smaller subgroups due to the large number of participants. Each facilitator led 8–9 members per group, and each session lasted 70–90 min. In each group session, each participant created a “Happiness Tree” drawing. One or two members shared their artwork, followed by group discussions on their experiences. Finally, the two subgroups merged into a larger group for collective reflection and experience sharing. To further ensure fidelity, the program followed a pre-defined structure and was implemented consistently across all sessions (see Table 1). Periodic consultations with the supervising expert helped address challenges encountered and supported adherence to the intervention protocol.

**Table 1:** Stages, stage objectives, and participant tasks in the “happiness tree” thematic art-based group counseling program.

Stage	Time	Stage objective	Participant tasks		
			Reporter	Group member	Group leader
Stage 1: Creating the “Happiness Tree”	30 min	Facilitate the integration of individual well-being resources through creative expression	Drawing the “Happiness Tree” as a Thematic Activity		Explanation of drawing instructions, maintaining time and order
Stage 2: Sharing the “Happiness Tree”	10 min	Promote emotionally safe expression and interpersonal sharing	Sharing personal happiness tree, introducing sources and related events.	Listen, understand, perceive, and feel the cases, without interrupting the reporter’s sharing.	Emphasize confidentiality; affirm the reporter’s sharing, and prohibit group members from giving judgments or suggestions; control the time.
Stage 3: Thematic Group Discussion	30-40 min	Deepen awareness of personal strengths and elicit resonant positive emotional experiences	Sit quietly, listen and feel, without participating in the discussion.	Combine the ‘Happiness Tree’ with personal experience, and speak and discuss around happiness resources. Do not evaluate others, only share your own feelings.	Encourage group members to speak freely; guide members to express their feelings by connecting their own ‘Happiness Tree’; pay attention to each member’s viewpoint, focusing on their emotional or physical sensations. Ensure the group is protected from being interrupted or criticized, and control the time.
Stage 4: Integration and Emotional Closure	10-15 min	Consolidate therapeutic gains and reinforce positive experiences	Listen and summarize the experience of this group activity in one sentence, expressing feelings.		Guide the group members back to the large group, listen, control the time, and express gratitude.

The counseling program was structured into three phases:

1. Phase 1 (session 1): group formation

- Introduced the goals and structure of group counseling.
- Emphasized confidentiality and established a safe group environment.
- Facilitated mutual understanding among participants.

1. Phase 2 (sessions 2–5): Group Growth and Maturation

- Participants engaged in self-reflection, creative expression, and group sharing.
- Promoted emotional resilience, self-awareness, and psychological flexibility.

1. Phase 3 (session 6): Conclusion and Reflection

- Addressed separation anxiety and closure.
- Provided opportunities for participants to share personal growth and insights.
- Facilitated future goal setting and self-development planning.

All group counseling sessions were conducted online. The detailed implementation process and participant tasks are summarized. The study involving human participants was reviewed and approved by the Department of Scientific Research and Teaching Affairs, Hefei Preschool Education College (approval date: April 26, 2023). Informed consent to participate in the study was provided to all participants.

### 3.4 Data analysis

Data were analyzed using SPSS version 24.0. All analyses followed the intention-to-treat (ITT) principle to preserve the benefits of randomization. For participants who discontinued the intervention after attending the first session and did not complete post-test assessments (n = 2 in the experimental group), missing data were imputed using the last-observation-carried-forward (LOCF) method. Continuous data were presented as median (Md) and interquartile range (IQR), as indicated in the tables. The Mann–Whitney U Test was employed to assess the homogeneity between the experimental and control groups, as well as to compare pre- and post-counseling differences. The Wilcoxon Signed-Rank Test was applied for within-group comparisons. Non-parametric effect sizes were expressed as rank-biserial correlations (*r*), with confidence intervals calculated using bootstrapping procedures in JASP version 19.3. Statistical significance was set at a *p*-value of less than 0.05.

## 4 Results

All results reported below are based on an ITT analysis, with missing post-test data for two participants in the experimental group imputed using the LOCF method. To evaluate the robustness of findings, a sensitivity analysis using a per-protocol approach was also conducted. The results yielded similar patterns of statistical significance and effect direction (see Supplementary Tables S1–S3).

### 4.1 Comparison of pre-test differences between the two groups

The Mann–Whitney U Test was performed to examine pre-test differences between the experimental and control groups. The results revealed no significant differences between the groups regarding mental health, subjective well-being, and general self-efficacy scores. These findings suggest that the two groups were homogeneous prior to the group counseling, thus validating the initial grouping based on positive psychological traits and justifying the continuation of the study (Table 2).

**Table 2:** Comparison of pre-counseling scores between experimental and control groups (Md, IQR).

Variable	Control group (*n* = 18)	Experimental group (*n* = 18)	*u* value	*p* value
Mental health	3.50 (8.00)	7.00 (5.50)	122.500	0.209
Subjective well-being	27.26 (7.68)	24.21 (6.91)	180.500	0.558
General self-efficacy	24.00 (5.00)	21.50 (8.50)	197.000	0.267

*p < 0.05; Values are reported as median (Interquartile Range), i.e., Md (IQR).

### 4.2 Within-group comparison of pre- and post-test scores

The Wilcoxon Signed-Rank Test was employed to compare pre- and post-test scores within the two groups. The results indicated a statistically significant increase in subjective well-being scores in the experimental group post-counseling compared to pre-counseling (Z = 116.000, *p* < 0.05). No significant changes were observed within the control group or in the experimental group’s scores for general self-efficacy, either pre- or post-counseling (Table 3).

**Table 3:** Within-group comparison of pre- and post-counseling scores in experimental and control groups (Md, IQR).

Variable	Group	Testing time		*z* value	*p* value
		Pre-test	Post-test		
Subjective well-being	Control	27.26 (7.68)	25.47 (6.58)	83.000	0.913
	Experimental	24.21 (6.91)	26.46 (5.68)	116.000*	0.013
General self-efficacy	Control	24.00 (5.00)	22.50 (7.00)	37.500	0.199
	Experimental	21.50 (8.50)	24.00 (5.50)	69.500	0.091

*p < 0.05; Values are reported as Median (Interquartile Range), i.e., Md (IQR); Results are based on ITT analysis with LOCF for handling missing data. Post-test scores for two experimental participants were imputed using this method.

### 4.3 Comparison of pre- and post-test score differences between the two groups

The Mann–Whitney U Test was utilized to compare the differences in pre- and post-test scores between the experimental and control groups. The results revealed that the experimental group exhibited significantly higher score differences in subjective well-being and general self-efficacy compared to the control group, with both differences reaching statistical significance (*U* subjective well-being = 78.500, *U* general self-efficacy = 80.000, both *p* < 0.05). In terms of effect sizes, the intervention yielded moderate effects on both subjective well-being (*r* = 0.404, 95% CI [0.046, 0.670] and general self-efficacy [*r* = 0.426, 95% CI (0.072, 0.684; Table 4)]. These effect sizes should be interpreted cautiously given small sample size and wide confidence intervals.

**Table 4:** Between-group comparison of change scores in subjective well-being and general self-efficacy (post–pre difference, Md, IQR).

Variable	Group	Post–Pre difference	*u* value	*p* value	Effect size (*r*)	95% CI for *r*	
						Lower bound	Upper bound
Subjective well-being	Control	−0.37 (3.86)	96.500*	0.038	0.404	0.046	0.670
	Experimental	1.95 (3.93)					
General self-efficacy	Control	−1.00 (4.00)	93.000*	0.028	0.426	0.072	0.684
	Experimental	2.00 (3.25)					

*p < 0.05; Values are reported as median (Interquartile Range), i.e., Md (IQR); Results are based on ITT analysis with LOCF for handling missing data. Post-test scores for two experimental participants were imputed using this method.

## 5 Discussion

The present study suggested preliminary improvements in subjective well-being and general self-efficacy among participants who received the “Happiness Tree” thematic art-based group counseling, compared to those in the control group. The results suggest that the “Happiness Tree” thematic art-based group counseling can serve as a potentially effective approach to enhancing the positive psychological traits of college students.

This study highlights the unique value of the “Happiness Tree” thematic art-based group counseling as an innovative intervention to promote positive psychological development among college students. Art functions not only as a medium for emotional expression but also as a powerful tool to foster positive emotional experiences. The symbolic design of the “Happiness Tree” helps participants visualize both internal and external sources of happiness through its components—roots, trunk, and crown. This process encourages individuals to focus on positive aspects of their lives, like personal strengths and social support, which contributes to the development of positive psychological traits. In addition, participants can learn from each other and experience more understanding, social support, and empathy in a supportive group environment. These interactions help deepen engagement. The behaviors developed in the group can be applied to various life contexts, such as school and family, which supports the continued development of positive psychological traits.

Our research shows that the “Happiness Tree” thematic art-based group counseling helped college students enhance their positive psychological traits particularly by improving subjective well-being and self-efficacy. This result matches previous studies showing that art therapy can improve subjective well-being and self-efficacy(Kaimal and Ray, 2016; Zhang et al., 2024). This may be due to the positive emotional experiences and the visualization of happiness resources during the painting process. Tree drawing and expression writing help participants find positive meaning in their daily events, benefiting both physical and mental health. Research on expression writing has confirmed that people can bring up positive emotions through recollection and writing(Ren et al., 2025; Zheng et al., 2019). During the “Happiness Tree” drawing exercise, participants reflected on positive events, support systems, and personal resources, which strengthened their awareness of life’s positive aspects, further enhancing subjective well-being. On the other hand, externalizing positive emotions through art provided a way for self-expression, which helped them reduce stress and enhance self-efficacy. Throughout this process, participants were able to recognize their progress and achievements, thus further boosting their positive psychological traits.

To further evaluate the practical significance of these improvements, within-group comparisons showed that the intervention group’s median score on subjective well-being increased by 2.25 points, and general self-efficacy increased by 2.50 points from pre- to post-intervention. According to a distribution-based approach, these score changes approximate the commonly accepted threshold of 0.5 standard deviations, which is often interpreted as a meaningful improvement (Norman et al., 2003). Together with the observed effect sizes (*r* subjective well-being = 0.404; *r* general self-efficacy = 0.426), while improvements approached conventional thresholds, they may reflect trends toward enhanced psychological functioning, though clinical significance remains uncertain without replication in larger studies.

The “Happiness Tree” thematic art-based drawing process strengthens participants’ internal experiences. Mutual understanding and support in group counseling provide a safe space for open expression, enhancing participants’ positive psychological traits. Sharing their artwork and discussing happiness resources and support systems allow individuals to receive positive feedback and recognition from others, thereby strengthening self-affirmation and self-awareness. In group interaction, participants become more aware of their strengths, which boosteds subjective well-being and self-efficacy, helping maintain optimal mental health(Bing et al., 2022; Caprara et al., 2022; Doménech et al., 2024; Xiyun et al., 2022). Additionally, through regular “Happiness Tree” drawing exercise, participants were encouraged to revisit their happiness resources, reinforcing their perception of happiness. This process fostered the gradual development of positive self-awareness, creating a long-term mechanism for enhancing positive psychological traits.

While this study confirmed the potential effectiveness of the “Happiness Tree” thematic art-based group counseling in improving the positive psychological traits of college students, several limitations should be acknowledged, particularly regarding its exploratory nature and statistical limitations. First, this study employed an online counseling format. Despite the advantages of online counseling in terms of time and flexibility, it presents certain challenges, such as prolonged group cohesion-building and weaker interpersonal connections among members (Kozlowski and Holmes, 2014). Although existing research has confirmed that both online and offline group counseling are effective when counseling protocols are consistent (Ierardi et al., 2022), exploring offline counseling models and comparing them with online counseling formats remains of practical significance. Future research should further investigate offline counseling models to enhance our understanding of how different formats contribute to therapeutic outcomes. Second, the small sample size in this study is a significant limitation that led to insufficient statistical power to detect medium effects. Specifically, post-hoc power analysis revealed that the achieved power was only 56.88% for subjective well-being and 62.38% for self-efficacy—both well below the commonly accepted threshold of 80%. This suggests that the study may not have been adequately powered to detect smaller effects, a limitation commonly observed in small-sample psychological intervention studies(Giner-Sorolla et al., 2024; White et al., 2019). Moreover, the sample was limited to university students from a specific region with significant gender imbalance (100% female in the experimental group), which may confound intervention effects as gender can influence responses to art-based interventions. The complete gender imbalance significantly limits interpretability and generalizability of findings, as research shows gender differences in art therapy responses(Geue et al., 2012; O’Donnell et al., 2022). Given these sample-related constraints, this study has been positioned as a pilot exploratory investigation. Accordingly, and in light of the small sample size, no multiple comparison corrections (e.g., Bonferroni adjustment) were applied. Future studies should recruit larger and more balanced samples, with a minimum of 80 participants per group, ensure diversity across different universities and academic disciplines, and consider gender-balanced recruitment to avoid gender-related confounds. Additionally, employing stratified randomization to reduce bias will further enhance the internal validity of the findings. These measures will increase the intervention’s generalizability to broader populations, making the results more applicable across different demographic groups. Third, the psychological profile of participants represents an additional limitation. All individuals were screened as psychologically healthy using the GHQ-20. It might have restricted the ability to assess the intervention’s therapeutic potential in individuals experiencing subclinical or clinical levels of distress. Future studies should consider including participants with mild to moderate emotional difficulties to more comprehensively evaluate the clinical utility of the “Happiness Tree” intervention. Fourth, this study assessed the effects based solely on a short-term intervention, without conducting long-term follow-up, which limits the ability to evaluate the sustained impact of the “Happiness Tree” thematic art-based group counseling. Future studies should incorporate longitudinal follow-up assessments at 1, 3, and 6 months post-intervention to better evaluate the durability of the intervention effects. As positive psychology interventions may show fade-out effects over time if not reinforced(Bailey et al., 2020; Hansen et al., 2021), such studies should also consider implementing booster sessions—such as structured positive psychology exercises, online expressive writing tasks, or brief group check-ins—to help maintain and consolidate psychological gains and support the sustained development of positive psychological traits. Fifth, this study employed a waitlist control group rather than an active control condition. While this design demonstrates the utility of the “Happiness Tree” intervention relative to no intervention, it cannot isolate specific therapeutic components from nonspecific factors. Prior research has highlighted that nonspecific factors—such as participant expectations or therapist attention—may also contribute to psychological improvements(Fernández-López et al., 2022). The lack of an active control group limits our ability to definitively attribute the observed effects solely to the intervention itself. Future studies should incorporate active control conditions, such as psychoeducational group sessions focused on the dissemination of positive psychology principles. Sixth, this study relied entirely on self-report instruments to evaluate psychological outcomes. Although the measures used were psychometrically sound and administered anonymously, the possibility of social desirability bias or culturally influenced response tendencies cannot be ruled out, particularly in a collectivist context such as China. Future studies should incorporate qualitative methods, including thematic analysis of participants’ artwork or post-session reflections, to gain deeper insights into the emotional and psychological processes underlying the intervention. Finally, while this study used self-efficacy and subjective well-being as indicators of positive psychological traits, it did not account for other factors that may influence these traits, such as emotional states and personality traits. Consequently, future research could consider incorporating more comprehensive psychological measures to further explore the relationship between these factors and positive psychological traits. Future studies should also explore the specific mechanisms of change in the “Happiness Tree” intervention. Understanding whether the therapeutic effects are driven by the drawing process, group sharing, individual reflection, or counselor support will be crucial for optimizing the intervention’s design and implementation. In particular, exploring the mediation pathways through which these therapeutic components (e.g., drawing, reflection, group sharing) influence the intervention’s outcomes will provide deeper insights into how specific elements of the intervention contribute to positive psychological changes.

In conclusion, the ‘Happiness Tree’ thematic art-based group counseling program warrants further investigation as an innovative approach for enhancing the positive psychological traits of college students. Its structured format, symbolic framework, and emphasis on group dynamics provide preliminary support for further development in art therapy research and practice.

## Data Availability

The original contributions presented in the study are included in the article/Supplementary material, further inquiries can be directed to the corresponding author/s.
