# Supplementary material for: “Happiness tree”: a thematic art-based group counseling method for enhancing positive psychological traits in Chinese college students
Source: Front Psychol. 2025 Aug 20;16:1651008. doi: 10.3389/fpsyg.2025.1651008 (PMC12406868; doi:10.3389/fpsyg.2025.1651008)
Supplement: Supplementary file 1 [file Data_Sheet_1.pdf]

frontiers

Supplementary Material：： Sensitivity Analyses (Per-Protocol vs. ITT)

Sensitivity Analyses Overview

To assess the robustness of the findings, we conducted a sensitivity analysis using a per-protocol (PP) approach, including only participants who completed both pre- and post-test assessments (n = 34). These results were compared to the primary intention-to-treat (ITT) analysis, which included all randomized participants (n = 36), with missing post-test values imputed using the last-observation-carried-forward (LOCF) method for two participants who discontinued after the first group counseling session. Although the results showed minor numerical differences between the two approaches, the overall patterns of significance and effect sizes remained similar, supporting the stability of the intervention's effects.

Table S1. Baseline Comparison Between Experimental and Control Groups (Md, IQR)

Variable	Control Group (n=18)	Experimental Group (n=16)	*U* Value	*P* Value
Mental Health	3.50(8.00)	7.00(5.75)	111.000	0.266
Subjective Well-Being	27.26(7.68)	24.21(6.15)	164.500	0.484
General Self-Efficacy	24.00(5.00)	21.50(9.25)	176.000	0.281

Table S2. Within-Group Comparison of Pre- and Post-Counseling Scores in Experimental and Control Groups (Md, IQR)

Variable	Group	Testing Time		*Z* Value	*P* Value
		Pre-test	Post-test		
Subjective Well-Being	Control	27.26(7.68)	25.47(6.58)	83.000	0.913
	Experimental	24.21(6.15)	26.46(4.88)	116.000*	0.013
General Self-Efficacy	Control	24.00(5.00)	22.50(7.00)	37.500	0.199
	Experimental	21.50(9.25)	24.00(5.75)	69.500	0.091

* *Note: P<0.05*

Table S3. Between-Group Comparison of Change Scores in Subjective Well-Being and General Self-Efficacy (Post–Pre Difference, Md, IQR)

**Variable**	**Group**	**Post–Pre Difference**	***U* Value**	***P* Value**	**Effect Size (*r*)**	**95% CI for *r***	
						**Lower bound**	**Upper bound**
**Subjective Well-Being**	Control	-0.37(3.86)	78.500*	0.022	0.455	0.096	0.709
	Experimental	2.34(4.35)					
**General Self-Efficacy**	Control	-1.00(4.00)	80.000*	0.027	0.444	0.083	0.703
	Experimental	3.00(3.75)					

**Note:* $P<0.05$
